# Validation of a New Liquid Asymmetric-Electrode Plasma Optical Emission Spectroscopy (LAEP-OES) Method for Measurement of Total Mercury in Tuna

**DOI:** 10.1093/jaoacint/qsae053

**Published:** 2024-06-28

**Authors:** Hidenori Takagi, Yoshiaki Shibuta, Michiaki Yamashita

**Affiliations:** ARKRAY, Inc., Research and Development Division, Yousuien-nai, 59 Gansuin-cho, Kamigyo-ku, Kyoto 602-0008, Japan; ARKRAY, Inc., Research and Development Division, Yousuien-nai, 59 Gansuin-cho, Kamigyo-ku, Kyoto 602-0008, Japan; Tokyo University of Marine Science and Technology, 4-5-7 Konan, Minato-ku, Tokyo 108-8477, Japan; National Fisheries University, 2-7-1 Nagata-Honmachi, Shimonoseki, Yamaguchi 759-6595, Japan

## Abstract

**Background:**

Mercury intake is caused by eating seafood, such as tuna and other predatory fish species. To reduce the health risks of mercury intake, it is necessary to continuously measure and monitor mercury concentrations at fish farms and markets. We have developed a compact system that can detect multiple heavy metals by liquid asymmetric-electrode plasma optical emission spectroscopy (LAEP-OES).

**Objective:**

The validity of the LAEP-OES method for total mercury levels was evaluated using standard solutions, certified substances, and specimens of bluefin tuna and other fish species.

**Methods:**

All specimens were dissolved in 4 M lithium hydroxide solution and then dispensed into a sample reservoir well of the single-use measurement reagent pack. Total mercury levels were automatically measured within 15 min of placement into the dedicated equipment. A total of 102 fish specimens, classified into 10 fish species, were evaluated using the new method and the results were compared to those obtained from validated analytical methods.

**Results:**

LOD (0.02 mg/kg), LOQ (0.07 mg/kg), repeatability (4.0%), intermediate precision (9.8%), and trueness (recoveries 107%) of the proposed method were within satisfactory limits for total mercury levels in fish. Additionally, when using various fish species, the method had a strong positive correlation with the results of cold-vapor atomic absorption spectrometry (CV-AAS, the official method) with Spearman *r_s_* = 0.984.

**Conclusion:**

The LAEP-OES method can be used for measuring total mercury levels in bluefin tuna. Total mercury measurement using this new method has the potential to be applied to other fish species.

**Highlights:**

Total mercury levels in fish were measured using our unique analysis system. Pacific bluefin tuna, southern bluefin tuna, and Atlantic bluefin tuna distributed in the Japanese market were analyzed for total mercury in their wild and farmed fish varieties.

Consumption of fish as a nutritious protein source is essential (1). However, large predatory fish, such as tuna and alfonsino, are known to contain a high amount of methylmercury due to bioaccumulation (2). There are three types of bluefin tuna that live in different ocean regions: Pacific bluefin tuna (*Thunnus orientalis; T. orientalis*), Southern bluefin tuna (*Thunnus maccoyii; T. maccoyii*), and Atlantic bluefin tuna (*Thunnus thynnus; T. thynnus*). Bluefin tuna is consumed in large quantities and can contain high amounts of methylmercury (3), which is a health risk for neurodevelopment of fetuses and children (4). Therefore, the recommended intake of tuna and other predatory fish, especially for the above populations, should follow the tolerable weekly intake set by the Codex Alimentarius Committee (5). It has been reported that about 60–99% of the total mercury in fish is methylmercury (6, 7). However, fish contain many nutrients that are beneficial, such as docosahexaenoic acid (DHA), eicosapentaenoic acid (EPA), omega-3 fatty acids, essential minerals, and vitamins (8). The American Heart Association recommends eating fish at least twice per week in order to reach the daily intake of omega-3 fatty acids (9). The Codex Alimentarius Committee continues to evaluate the risks and benefits of consuming fish (10). Based on discussions of standards for total mercury or methylmercury contained in fish by the Codex Alimentarius Committee (11), each country or region has established its own standards for total mercury or methylmercury contained in fish. If the mercury level exceeds the regulatory limit, sales may be suspended or a product recall could be mandated depending on the country or region, resulting in financial losses to the sales company (12, 13). Such problems can be avoided if mercury testing is performed before export or sale. Additionally, if the mercury content of fish could be controlled at the production site, consumers would receive safer food products. However, many fishing grounds and aquaculture farms do not have the capacity to test for heavy metals on-site and commercial testing takes nearly a month to obtain results.

There are various methods for measuring heavy metals in foods. Generally, total mercury and methylmercury are determined using government-defined methods or methods certified by organizations such as AOAC International. Cold-vapor atomic absorption spectrometry (CV-AAS) (14) and GC with an electron capture detector (GC–ECD; 15) are used for detecting mercury and methylmercury, respectively. These methods require the use of multiple acidic solutions and organic solvents, as well as centrifugation and high-temperature treatment. Furthermore, the detector requires appropriate measurement conditions to be set, which is complicated. Cold-vapor atomic fluorescence spectrometry (CV-AFS; 16) requires various acids, hydrogen peroxide (H_2_O_2_), sodium hydroxide (NaOH), sodium borohydride (NaBH_4_), and a carrier gas. AMA-254 (17) and DMA-80 (18) systems measure mercury using thermal-vaporization atomic absorption spectrometry. These systems require oxygen for drying and thermally decomposing specimens, an amalgam for selectively trapping the mercury vapor and two types of optical cells with different cell lengths for measuring mercury concentration. The optical cells, the amalgam and other system components deteriorate over time, as does the light source, necessitating periodic maintenance. Inductively coupled plasma-optical emission spectroscopy (ICP-OES; 19) and inductively coupled plasma-mass spectrometry (ICP-MS; 20) are typical methods for measuring multiple metals in various specimens (21). To measure multiple metal species simultaneously, HPLC can be combined with ICP-OES (22) or ICP-MS (23). Liquid electrode plasma optical emission spectroscopy (LEP-OES) is also a novel analysis method (24, 25). This method uses a narrow channel between Pt electrodes in which plasma and atomic excitation occur upon application of high direct voltage (800–1500 V). The plasma emission occurs within a narrow channel. When the concentration of metal ions is low, since there is no method for concentrating metal ions in the microchannel, a separate concentration operation is required. Measurements are made after extracting, purifying, and concentrating heavy metals from foods. However, the above analysis methods require complex, expensive systems and specialized knowledge.

Herein, a unique analysis system was developed for use by non-specialists that has the potential to easily measure heavy metals on-site. The system consists of the SillBe Kit Hg, Pb (ARKRAY, Inc., Kyoto, Japan), which includes a single-use measurement reagent pack with a plasma light-emitting well that can concentrate metal ions near a fine electrode and generate plasma, and the SillBe LB-5410 (ARKRAY, Inc.), which is an automatic measuring device consisting of a spectrometer and a detector for the SillBe Kit Hg, Pb (Supplemental Figure 1). The key difference between this method and that used by existing LEP-OES devices is that the plasma is generated on a fine electrode based on the differences in electrode size and electrode material (asymmetric electrode). Therefore, in this study, to distinguish this method from LEP, we refer to it as liquid asymmetric-electrode plasma optical emission spectroscopy (LAEP-OES). It is indeed imperative to comprehend the precise position of plasma formation for these two methods. To illustrate, a prototypical LEP configuration employs platinum (Pt) electrodes positioned on either side of a narrow channel (24). Upon application of an electrical potential to the Pt electrodes immersed in a liquid medium, plasma generation is efficiently induced within this microchannel [Figure 1(a)]. In contrast, the LAEP approach eschews the utilization of such narrow channels. It instead utilizes electrodes with different sizes and made of different materials (nichrome and carbon), with plasma generation occurring predominantly at the location of the smaller electrode [Figure 1(b)]. A notable characteristic of this approach is its capacity to enhance the concentration of metal ions at the electrode sites responsible for plasma generation prior to the actual initiation of plasma. This represents a capability conspicuously absent in LEP-OES. This method predominantly causes metal ion accumulation near the electrode, thus intensifying the emission peaks due to metal species in the plasma. Consequently, this facilitates the detection of trace elements in a test specimen. Furthermore, it eliminates the need for purification and concentration steps, which can shorten measurement times. The concentration of heavy metals can be measured simply by placing the alkali-treated fish specimen and a single-use measurement reagent pack in the instrument. In this study, the validity of the proposed LAEP-OES method for measuring total mercury in fish was examined. In total, 102 fish specimens, classified into 10 fish species, were evaluated using validated analytical methods. The results were compared with the common official method in Japan for mercury measurement, known as CV-AAS [Figure 1(c)].

**Figure 1.**
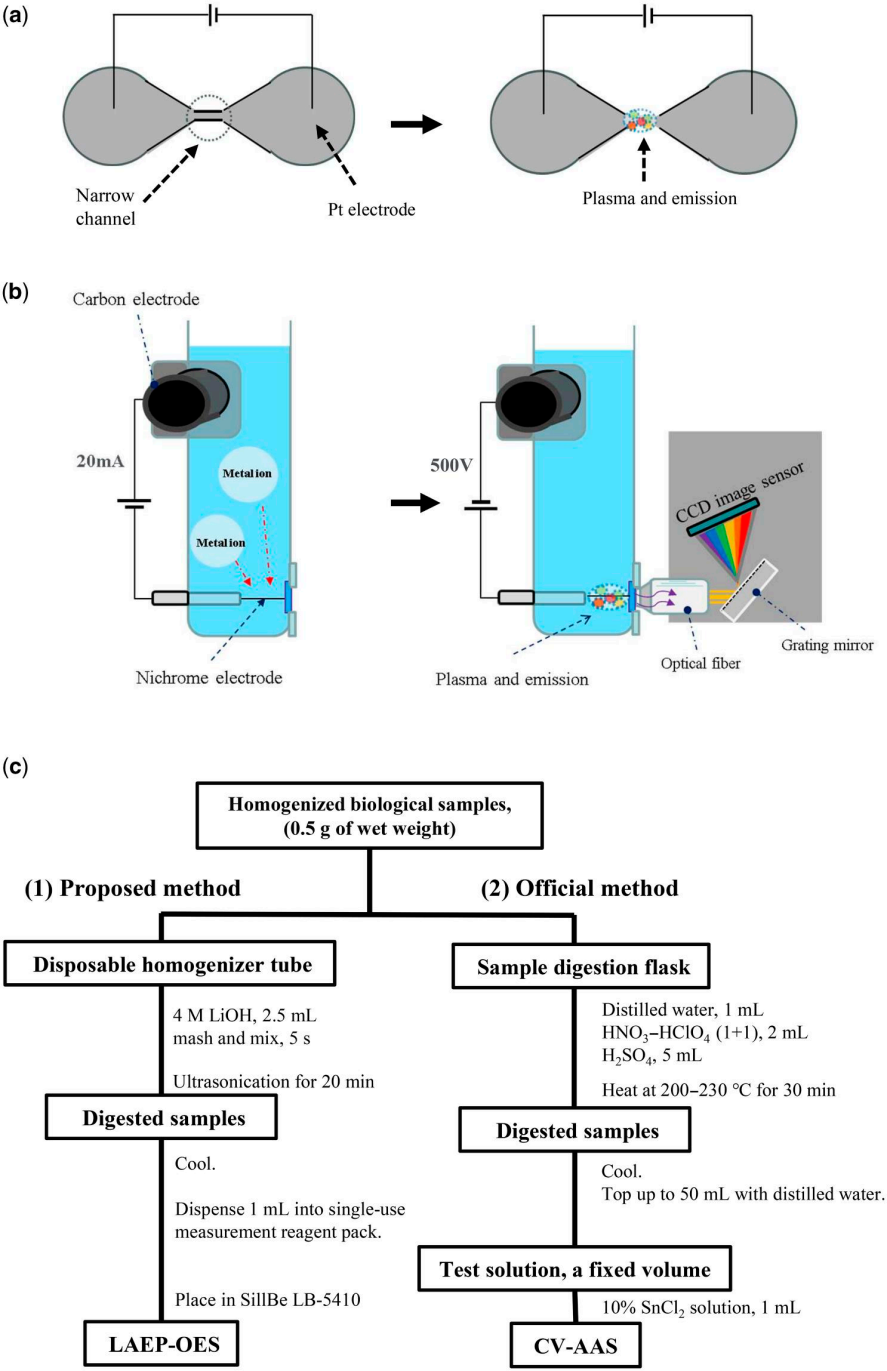
Comparison of LAEP and LEP principles, and LAEP-OES versus CV-AAS procedures. (a) Principle of LEP (24). A prototypical LEP configuration employs platinum (Pt) electrodes positioned on either side of a narrow channel. Upon application of an electrical potential to the platinum electrodes immersed in a liquid medium, plasma generation is efficiently induced within this narrow channel. (b) Principle of LAEP. The LAEP configuration utilizes electrodes with different sizes and made of different materials (nichrome and carbon), with plasma generation occurring predominantly at the location of the smaller electrode. A notable characteristic of this approach is its capacity to enhance the concentration of metal ions at the electrode sites responsible for plasma generation prior to the actual initiation of plasma. After excitation and light emission of metal atoms, the emitted light is introduced into an optical fiber. Its spectrum is obtained using a grating mirror and then measured using a charge-coupled device (CCD) image sensor. The built-in spectroscope measures the light generated by the excited metal atoms as a luminescence spectrum. (c) (I) Flowchart for the LAEP-OES method. A solution of 4 M lithium hydroxide (4 M LiOH) was used for extraction of mercury from fish. (II) Flowchart for the CV-AAS method, which is the method for total mercury measurement based on the guidelines of the Ministry of the Environment in Japan (14). Nitric acid (HNO_3_), perchloric acid (HClO_4_), and sulfuric acid (H_2_SO_4_) were used to treat the specimens in this method.

## Experimental

### Samples

- *Certified standard material.—*ERM^®^-BB442 (fish protein) was used as a reference material.
- *Fish specimens (102 in total).—*Approximately 200 to 300 g of frozen fish products in a vacuum pack were purchased between 2022 and 2023 from 42 sales companies using 47 electronic commerce sites in Japan: 25 *Thunnus orientalis* (Pacific bluefin tuna), 29 *Thunnus maccoyii* (Southern bluefin tuna), 27 *Thunnus thynnus* (Atlantic bluefin tuna), 3 young *Thunnus orientalis* (young Pacific bluefin tuna), 4 *Thunnus alalunga* (albacore tuna), 7 *Thunnus albacares* (yellowfin tuna), 4 *Thunnus obesus* (big eye tuna), 1 *Sardinops melanostictus* (Japanese sardine), 1 *Berardius bairdii* (Baird’s beaked whale), and 1 *xiphias gladius* (swordfish).

### Apparatus

- Food processor (MK-K82-W/Panasonic, Tokyo, Japan).
- Sartorius A120S analytical balance (Mettler-Toledo International, Inc., Columbus, OH, United States).
- Ultrasonic cleaner-dual frequency, ASU-10D: High, 23 kHz, 20 min (As One, Osaka, Japan).
- BioMasher^®^ SP; disposable homogenizer tube, 15 mL size (Nippi, Inc., Tokyo, Japan)
- Power Masher II (Nippi, Inc.).
- CV-AAS instrument; semi-automated mercury analyzer (Hg-201, Sanso Seisakusho Co., Ltd, Tokyo, Japan).
- Unipack (D-8 120 × 8 Unipack 5 mm 0.08 mm/D-8/SEISAN NIPPONSHA, Ltd, Tokyo, Japan).
- Biomedical freezer (MDF-U538D, SANYO Electric Co., Ltd, Osaka, Japan).
- Medical refrigerator (NC-ME100EC, Nihon Freezer Co., Ltd, Tokyo, Japan).
- SillBe LB-5410 (ARKRAY, Inc.). An automated metal analyzer consisting of a spectrometer and detector, which is a device developed to detect heavy metals (mercury and lead) in human urine (26). It also measures creatinine in urine and can correct for metal concentrations. Spectral data can be extracted using special software. In this study, the program was partially modified and adapted for fish. The mercury measurement time after placement in the device was 15 min. The device is small at 320 mm (width) × 400 mm (depth) × 430 mm (height) and 25 kg weight and can conduct measurements on-site [Supplemental Figure 1(a)].

### Reagents and Standards

- Distilled water (Yamato Scientific Co., Tokyo, Japan) was used throughout this study.
- Lithium hydroxide solution, 4 mol/L (Fujifilm Wako Pure Chemicals Corporation, Osaka, Japan)
- Methylmercury (II) chloride, standard solution in H_2_O (Thermo Scientific Chemicals, United States)
- Mercury standard solution (Fujifilm Wako Pure Chemicals Corporation)
- SillBe Kit Hg, Pb (ARKRAY, Inc.). The kit includes supplies for six measurements: six single-use reagent packs for measuring heavy metals (mercury and lead) in human urine, seven single-use droppers, and seven single-use tips for use with a SillBe LB-5410 nozzle [Supplemental Figure 1(b)]. The single-use heavy metal measurement reagent pack has 10 wells that have various roles for detecting heavy metals in urine, including a sample reservoir well, a dilution well, a protein removal well, a protein measurement well, five reagent storage wells (each storing a different reagent), and a plasma light-emitting well [Supplemental Figure 1(c)]. The light-emitting well concentrates metal ions near a fine electrode and allows LAEP-OES to be performed. When a test specimen is added to the sample reservoir well of this reagent pack and the pack is inserted into the SillBe LB-5410, clicking the “Start” icon displayed on the screen will initiate the process. Once started, the measurement occurs automatically. In this study, the sequencing program of the SillBe LB-5410 was partially modified for fish, such that not all wells of the pack were used (details are described in *Total Mercury—the LAEP-OES Method*).

### Sample Preparation

Approximately 200 to 300 g of frozen fillet of fish in a vacuum pack were thawed in ice water. After thawing, the fish was cut into three to four blocks, placed in a food processor, and thoroughly homogenized. Thereafter, 50 g to 70 g of each specimen were placed in a Unipack and placed in a −30°C freezer. The portion to be measured the next day was stored in a refrigerator at 4°C.

### Total Mercury—the LAEP-OES Method

- *Alkaline treatment of fish samples and certified standard material.—*After the balance was calibrated, a minced fish specimen weighing 0.5 g was measured with a precision of ±0.0009 g for total mercury analysis. The test specimen was added to a BioMasher SP, which is a disposable homogenizer tube. The test specimen in the tube was soaked in 2.5 mL 4 mol/L lithium hydroxide solution (4 M LiOH). The certified standard material ERM-BB442, a dry powder consisting of fish protein, was used for total mercury measurements; reports indicate that fish protein constitutes approximately 20% of total fish mass (27). Accordingly, we used 0.1 g powder, which represents 20% of a 0.5 g specimen, for measurements with 2.5 mL 4 M LiOH. For the LAEP-OES method, which typically requires 0.5 g of specimen, the measurement value obtained from 0.1 g ± 0.0009 g of the material was multiplied by 5 to extrapolate the results. We used a minimum required amount of 0.2 g ± 0.009 g of the certified material in combination with 5 mL 4 M LiOH. Next, the fish test specimen or ERM-BB442 test specimen was mashed and mixed using a masher rod connected to the Power Masher II for approximately 5 s. The tube was placed into a rack installed in the tank of an ultrasonic cleaner. The water temperature in the ultrasonic bath was around 20–25 °C and the oscillation conditions of the ultrasonic bath were as follows: high setting, 23 kHz for 20 min. After the ultrasonic treatment, the tube was removed from the ultrasonic bath and the test specimen was stirred well using the masher rod. The test specimen was allowed to stand until the temperature returned to room temperature. Finally, the masher rod was removed from the tube. For each alkali-treated fish specimen, a fresh single-use measurement reagent pack equipped with a plasma light-emitting well was utilized.
- *Specimen measurement using SillBe LB-5410.—*Using a single-use dropper, approximately 1 mL of the alkali-treated fish specimen was dispensed into a sample reservoir well of the single-use measurement reagent pack, which includes a light emission well, provided in the SillBe Kit Hg, Pb. The LAEP-OES method was used for the measurement. For measuring the mercury concentration of fish, the sequencing program was partially modified for fish. The single-use measurement reagent pack was then placed in the SillBe LB-5410. A single-use tip for the SillBe LB-5410 nozzles, which are used for dispensing solutions, was placed in the SillBeLB-5410. To initiate the process, the “Start” icon displayed on the screen of the SillBe LB-5410 was clicked. All subsequent steps were performed automatically at room temperature as follows.
  The 238 μL alkali-treated fish specimen in the reservoir well was transferred to other wells for dilution with 4.4 M LiOH (195 μL), 86 mM thallium(I) nitrate solution (21.6 μL), and ethanol (EtOH; 45.5 μL). These reagents are packed in each storage well of the single-use measurement reagent pack. After mixing, the diluted specimen was transferred into a light-emitting well in which electrodes were placed. A carbon electrode (ø4.0 mm, area in contact with liquid ≥33 mm^2^) and a nichrome electrode (ø0.1 mm × 0.5 mm) were used. A constant current of 20 mA for 180 s for metal enrichment and a direct current pulse voltage of 500 V (50 μs cycle, 50 times) for plasma emission were used [Figure 1(b)]. Mercury and thallium luminescence were measured simultaneously. The plasma emission sequence, which includes the metal ion concentration process and the plasma emission process, was performed twice. The average mercury concentration was calculated using Equation (1), based on the mercury and thallium luminescence intensity measurements, and the results from the two measurements were averaged. For each plasma emission sequence, plasma emission data for 2.5 ms (50 μs × 50 times) were acquired.
- *Calculation of total mercury value from the LAEP-OES method.—* Using the emission signal intensity at a specific wavelength obtained from the alkali-treated fish specimen  [Supplemental Figure 1(d)], the mercury concentration was calculated. The mercury ratio was calculated by using the ratio of the signal intensity at the mercury-specific wavelength to the baseline signal intensity and subtracting 1 (mercury signal intensity at 253.7 nm/baseline intensity −1). Similarly, for thallium added as an internal standard, the ratio of the signal intensity at the thallium-specific wavelength to the baseline signal intensity was calculated and 1 was subtracted (thallium signal intensity at 276.8 nm/baseline intensity −1) as in Equation (1) below. Finally, the ratio of the mercury ratio to the thallium ratio was used for the mercury concentration.
  (1) $$\mathrm{Value}=(\frac{Hg\;\mathrm{signal}\;\mathrm{intensity}}{Hg\;\mathrm{baseline}\;\mathrm{intensity}}-1)/(\frac{Tl\;\mathrm{signal}\;\mathrm{intensity}}{Tl\;\mathrm{baseline}\;\mathrm{intensity}}-1)$$

### Total Mercury Analysis—CV-AAS Method

The frozen fish specimens were sent to a chemical analysis laboratory (IDEA Consultants, Inc., Tokyo, Japan) for total mercury measurements based on the guidelines of the Ministry of the Environment in Japan (14).

### Calibration Curves for LAEP-OES Method

Preparation of 0, 0.06, 0.12, 0.6, 1.2, and 1.8 mg/kg methylmercury standard solutions was performed by dilution of a 1000 mg/kg methylmercury solution with 4 M LiOH to create a calibration curve. Since the concentration is calculated based on the emission wavelength for mercury, the adjusted mercury concentrations are 0, 0.048, 0.096, 0.48, 0.96, and 1.44 mg/kg, respectively, when considering the molecular weight of mercury in methylmercury. Next, approximately 1 mL of a specimen with the desired concentration was dispensed into a sample reservoir of the single-use measurement reagent pack of the SillBe Kit Hg, Pb. The kit was then placed in the SillBe LB-5410. To initiate the process, the “Start” icon displayed on the screen of the SillBe LB-5410 was clicked. All subsequent steps were performed automatically. Six concentrations of methylmercury were measured at random three times each. Eighteen single-use measurement reagent packs were used.

### Statistical Analysis

The Shapiro–Wilk test was used to test the normality for all data. Pearson’s correlation coefficient and linear regression were used to analyze the calibration curves for the LAEP-OES method. One-way analysis of variance (ANOVA) was used to analyze repeatability and intermediate precision for mercury detection using the LAEP-OES method. The Mann–Whitney test was used to analyze the differences in the median total mercury levels in the wild and farmed tuna. Spearman’s rank correlation coefficient was used to analyze the correlation between the results obtained by the LAEP-OES and CV-AAS methods. The Wilcoxon matched-pairs signed rank test was used to compare the median total mercury levels for the LAEP-OES and CV-AAS methods. Two-tailed *P* values were determined, and *P *<0.05 was considered to indicate statistical significance. All statistical analyses were performed using Prism (version 10; GraphPad, San Diego, CA, United States).

## Results

### Flowcharts for Determination of Total Mercury in Fish

**Figure 2.**
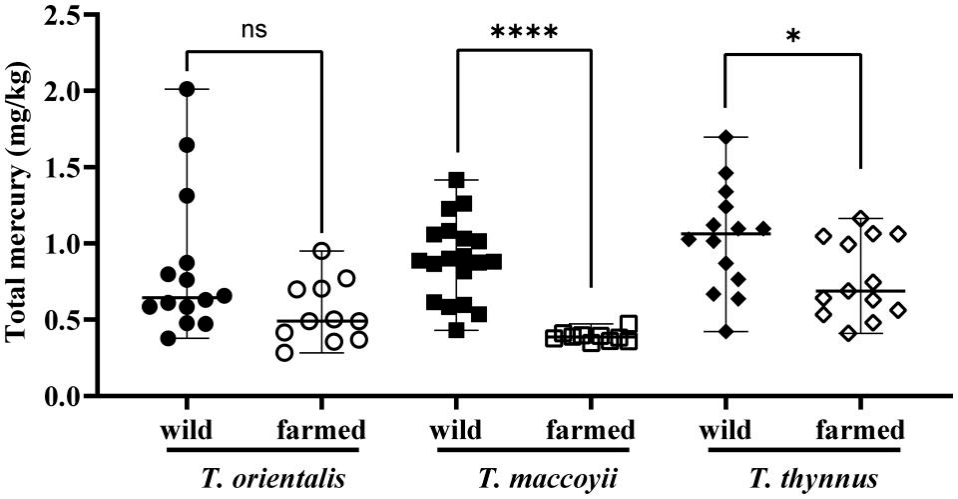
Differences in mercury concentration between wild and farmed Pacific, Southern and Atlantic bluefin tuna. Three types of bluefin tuna—Pacific bluefin tuna *(T. orientalis*), Southern bluefin tuna (*T. maccoyii*), and Atlantic bluefin tuna (*T. thynnus*)—are each plotted for farmed and wild-caught types. The middle line indicates the mean value, and the upper and lower lines indicate the maximum and minimum, respectively. A significant difference in mercury concentration between wild and farmed tuna was observed in *T. maccoyii* and *T. thynnus*. The Mann–Whitney test was used. *P <0.05; **P <0.01; ***P <0.001; ****P <0.0001; ns = not significant.

Flowcharts for measuring total mercury using our proposed method and the CV-AAS method are shown in Figure 1(c)-1 and (c)-2, respectively. Compared to the CV-AAS method, the LAEP-OES method in this study required fewer types of reagents for pretreatment.

### Validation Results

**Figure 3.**
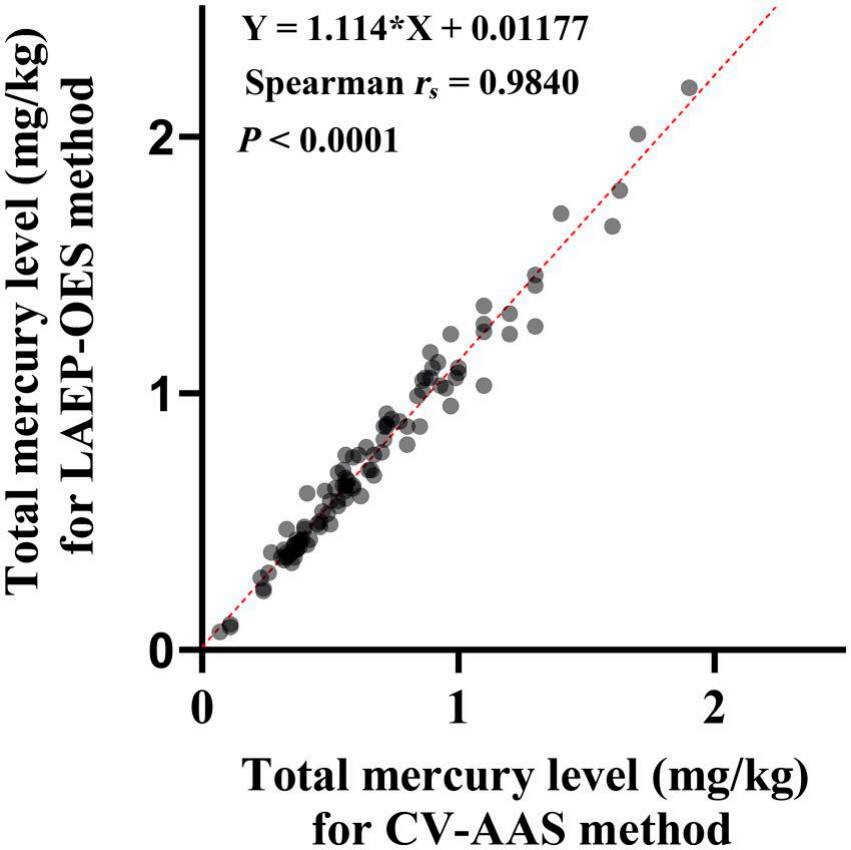
Comparison of total mercury concentration between the two methods. The correlation analysis was performed by plotting the mercury concentration in fish determined by the LAEP-OES and CV-AAS methods on the *y*- and *x*-axis, respectively. The correlation between the total mercury value obtained by LAEP-OES and CV-AAS was significant (Spearman’s rank correlation coefficient, *r_s_* = 0.9840, *P *<0.0001). A simple linear regression is shown as a dashed line.

- *Linearity of mercury levels using standard solutions in the LAEP-OES method.—*To create a calibration curve, six mercury solutions of varying concentrations were prepared using a methylmercury standard solution. The calibration curve exhibited linearity in a mercury concentration range of 0–1.5 mg/kg. Linear regression was used to calculate the mercury concentrations (*y*-axis: value obtained by LAEP-OES method; *x*-axis: mercury concentration; slope = 0.6100; *y*-intercept = −0.003889; Pearson r = 0.9998; R^2^ = 0.9961; *P *<0.0001, Supplemental Figure 2).
- *Recovery rates and selectivity for mercury in the LAEP-OES method.—*To evaluate whether the LAEP-OES method can detect inorganic mercury or methylmercury in fish, recovery tests were conducted by spiking 0.5 g minced fish with 300 ng mercury standard solution or methylmercury standard solution. The total mercury concentrations for young *T. orientalis* (young Pacific bluefin tuna) and *T. thynnus* were 0.1 and 0.69 mg/kg, respectively, before spiking. Both methylmercury and inorganic mercury were detected with marginal recovery rates in the range of 90–104% (Table 1).
- *LOD, LOQ, accuracy, and stability for mercury detection using the LAEP-OES method.—*To evaluate the accuracy and stability of the measurement method for total mercury in a fish matrix, ERM-BB422 (fish protein) was analyzed for validation of the LAEP-OES method (Table 2). Following the guidelines of the Ministry of Health, Labor, and Welfare of Japan for the validation of methods for metal analysis in food (28), measurements were conducted using duplicate analyses separated by 5 days. The repeatability relative standard deviation (RSD_r_) and relative standard deviation for intermediate precision (RSD_i_) were 4.0 and 9.8%, respectively. The LOD and LOQ for the LAEP-OES method were 0.02 and 0.07 mg/kg, respectively. The mercury concentration in ERM-BB422 was 0.601 ± 0.030 mg/kg, whereas that determined using the proposed method was 0.645 ± 0.060 mg/kg. The average value of the mercury concentration was about 7% higher than that of the certified materials. These results satisfied the AOAC *Standard Method Performance Requirements* (29).
- *Applicability of the LAEP-OES method.—*To investigate the measurement range required for measuring the total mercury content in fish, a survey of guidelines for mercury concentration in fish in different countries and regions was conducted (30–39). The Codex Alimentarius Commission has adopted a guideline level of 0.5 mg/kg methylmercury for fish excluding predatory fish and 1.0 mg/kg methylmercury for predatory fish (11). The guidelines differ depending on the country and region (Table 3). Regarding predatory fish, Hong Kong had the strictest regulations (0.5 mg/kg). Most countries had a standard of 1 or 0.5 mg/kg for total mercury or methylmercury. These guideline levels were within the detectable range of the LAEP-OES method.
- *Investigation of the range of total mercury content in commercially available bluefin tuna.—*To investigate the distribution of mercury values in bluefin tuna on the market, and to evaluate the difference in mercury concentrations between wild and farmed tuna, 81 tuna specimens among three types of bluefin tuna were investigated (Table 4 and Figure 2). The total mercury concentration in all specimens could be measured using the LAEP-OES method. Significant differences in mercury concentration between wild and farmed tuna were observed in *T. maccoyii* and *T. thynnus* (Mann–Whitney test, *P *<0.0001 and *P *<0.05, respectively). Additionally, wild bluefin tuna tended to have a wider range of concentrations than farmed bluefin tuna.
- *Comparison of LAEP-OES and CV-AAS methods.—*To evaluate the total mercury measurement performance of the LAEP-OES method, a comparison analysis was performed with CV-AAS using a variety of specimens (Figure 3, Supplemental Table 1). The mercury levels in various fish specimens could be detected using the LAEP-OES method (Table 5). The data for each method were not normally distributed (LAEP-OES method, CV-AAS method, Shapiro–Wilk test, *P <*0.0001). A high correlation was observed between the LAEP-OES and CV-AAS methods (Spearman’s rank correlation coefficient, *r_s_* = 0.9840, *P *<0.0001, Figure 3). The slope of the simple linear regression line was greater than unity. Overall, the median value was higher for the LAEP-OES method than for the CV-AAS method (0.67 versus 0.57, respectively, Wilcoxon matched-pairs signed rank test, *P *<0.0001).

**Table 1.** Marginal recovery rates^a^ by spike-in test

Specimen name	Methylmercury (CH_3_Hg^+^)		Inorganic mercury （Hg^2+^）		
	Recovery, %	SD, %	Recovery, %	SD, %	N^b^
Young *T. orientalis*	95	1.1	90	6.0	3
*T. thynnus*	104	6.5	96	4.9	3

a Marginal recovery rate, % = (W_1_-W_2_)/W_0_ × 100. W_1_ represents the analytical value obtained for the spiked specimen, W_2_ represents the analytical value obtained for the unspiked specimen, and W_0_ represents the spiking amount.

b N = number of specimens.

**Table 2.** LOD, LOQ, accuracy, and stability for mercury detection^a^

Specimen name	LOD, mg/kg	LOQ, mg/kg	RSD_r_, %	RSD_i_, %	Trueness, %	N^b^
ERM-BB422	0.02	0.07	4.0	9.8	107	10

a Measurements were conducted using duplicate analyses separated by 5 days.

b N = number of specimens.

**Table 3.** Guideline levels for mercury in fish by region and country

Region and country	All fish and their products except predatory fish	Predatory fish, specific fish	Ref.
Australia, New Zealand	Total mercury: 0.5 mg/kg	Total mercury: 1 mg/kg	(30)
Canada	Total mercury: 0.5 mg/kg	Total mercury: 1 mg/kg	(31)
China	Methylmercury: 0.5 mg/kg	Methylmercury: 1 mg/kg or 1.2 mg–1.7 mg/kg, depending on the specific type of fish; for example, tuna at 1.2 mg/kg	(32)
European Union	Total mercury: 0.3 mg/kg or 0.5 mg/kg, depending on the specific type of fish; for example, anchovy at 0.3 mg/kg	Total mercury: 1 mg/kg	(33)
Hong Kong	Methylmercury: 0.5 mg/kg		(34)
Japan	Total mercury: 0.4 mg/kg	Not applicable	(35)
	Methylmercury: 0.3 mg/kg		
Singapore	Total mercury: 0.5 mg/kg	Total mercury: 1 mg/kg	(36)
South Korea	Total mercury: 0.5 mg/kg	Methylmercury: 1 mg/kg	(37)
Taiwan	Methylmercury: 0.5 mg/kg	Methylmercury: 1 mg/kg or 2 mg/kg, depending on the specific type of fish; for example, tuna at 2 mg/kg	(38)
United States	Methylmercury: 1 mg/kg		(39)

**Table 4.** Comparison of total mercury content between wild and farmed bluefin tuna

Specimen name	Type	N^a^	Minimum, mg/kg	25% Percentile, mg/kg	Median, mg/kg	75% Percentile, mg/kg	Maximum, mg/kg	Range, mg/kg
*T. orientalis*	Wild	14	0.38	0.56	0.64	0.98	2.01	1.63
	Farmed	11	0.28	0.37	0.49	0.70	0.95	0.67
*T. maccoyii*	Wild	19	0.43	0.62	0.89	1.06	1.42	0.99
	Farmed	10	0.35	0.36	0.39	0.40	0.47	0.12
*T. thynnus*	Wild	14	0.42	0.74	1.06	1.27	1.70	1.27
	Farmed	13	0.41	0.55	0.69	1.05	1.16	0.75

a N = number of specimens.

**Table 5.** Total mercury in edible fish parts determined using the LAEP-OES method

Specimen name	N^a^	Minimum, mg/kg	25% Percentile, mg/kg	Median, mg/kg	75% Percentile, mg/kg	Maximum, mg/kg	Range, mg/kg
*Thunnus orientalis*	25	0.28	0.48	0.61	0.79	2.01	1.73
*Thunnus maccoyii*	29	0.35	0.40	0.62	0.97	1.42	1.07
*Thunnus thynnus*	27	0.41	0.64	0.99	1.10	1.70	1.23
young *Thunnus orientalis*	3	0.09	0.09	0.10	0.3	0.30	0.21
*Thunnus alalunga*	4	0.34	0.35	0.40	0.42	0.43	0.09
*Thunnus albacares*	7	0.23	0.24	0.64	0.70	1.80	1.57
*Thunnus obesus*	4	0.62	0.66	1.01	1.26	1.27	0.65
*Sardinops melanostictus*	1	—^b^	—	0.07	—	—	—
*Xiphias gladius*	1	—	—	0.76	—	—	—
*Berardius bairdii*	1	—	—	2.19	—	—	—
Overall	102	0.07	0.43	0.67	1.02	2.19	2.12

a N = Number of specimens.

^b^— = Not applicable due to an insufficient number of specimens.

## Discussion

To evaluate the performance of the LAEP-OES method for mercury detection in fish, certified materials were used and the results were compared with the official method using 102 fish specimens (Figure 1). The LAEP-OES method utilizes thallium nitrate as an internal standard to measure heavy metals more accurately. The internal standard method is an analysis method in which the luminescence intensities for the measurement element and the internal standard element are measured simultaneously, and the concentration is calculated using the ratio of the luminescence intensities. Thus, it is possible to correct not only for fluctuations in light emission intensity caused by physical effects such as plasma fluctuations, but also dimensional errors in the light-emitting cells. Using the single-use heavy metal measurement reagent pack that has all the necessary reagents and LAEP-OES, total mercury measurements can be performed by simply dispensing the specimen solution (Supplemental Figure 1).

Sample volume and chelating agents are factors that can affect measurement accuracy in chemical assays. Specifically, adding a sample mass greater than 0.75 g to 2.5 mL 4 M LiOH can introduce variability and reduce the expected measurement outcome. Conversely, the use of chelating agents, which are known to sequester heavy metals, enhances heavy metal peak signals since chelating agents can enhance the detection sensitivity. However, in the current study, chelating agents were not needed for the methodology since mercury levels could be directly assessed in tuna samples.

Since methylmercury is mainly contained in tuna, a methylmercury standard solution was used for creating the calibration curve. To confirm whether the method could detect inorganic mercury or methylmercury in fish, several spike-in tests were conducted. When measuring specimens by adding methylmercury or inorganic mercury solutions to minced fish, high recovery rates were observed for both methods. Therefore, this method can effectively measure total mercury (Table 1). To test the possibility of measuring other metals, unique peaks for cadmium and lead were observed in tests in which standard solutions of cadmium and lead were added to fish. However, in this study, method validation was not performed for bluefin tuna because past reports have shown that it is low in cadmium and lead (40, 41). In the future, the validation could be performed according to regulation needs.

The performance of the total mercury analysis method was evaluated through repeated analysis of a certified standard material (Table 2). RSD_r_ and RSD_i_ were 4.0 and 9.8%, respectively. The mercury concentration of the ERM-BB-422 specimen was 0.601 ± 0.030 mg/kg; the trueness was 107% (0.645 ± 0.060 mg/kg). The LOD and LOQ for the LAEP-OES method were 0.02 and 0.07 mg/kg, respectively. To investigate the measurement range required for measuring total mercury in fish, a survey of the current guideline levels (GLs) for methylmercury in fish was conducted (Table 3). According to the guidelines (28, 42) for the measurement method, the appropriate range for measuring mercury in fish is 1 of 10 GLs, i.e., two times GLs (0.1–2 mg/kg). Therefore, it was considered that the LAEP-OES method can be used for fish measurements.

To determine whether the LAEP-OES method can be utilized to investigate the total mercury content of bluefin tuna that is distributed, the number of specimens was increased (Table 4). This survey reinforced the validity of the LAEP-OES method for measuring total mercury in bluefin tuna. However, sufficient information on bluefin tuna was not obtained for a full understanding of the results, i.e., the weight of the fish, how old it was, and whether it was fully farmed or raised. In order to gain deeper knowledge, it is necessary to obtain the tuna fish body rather than just a portion. Interestingly, all types of wild tuna tended to have a wider range of mercury concentrations than farmed tuna. The narrow range of mercury levels in farmed bluefin tuna is thought to be due to the fact that mercury levels can be controlled by feeding methods (43). Since some farmed tuna can have high mercury levels, a method that can monitor and display mercury levels in advance is necessary for food safety (Table 4 and Figure 2). While there are concerns about the possibility of reputational damage due to the display of mercury concentrations on fish, a group that was presented with information on regulatory values that take into account a 10-fold safety factor had a significantly higher sense of safety than a group that was not presented with this information (44). It is thought that a sense of security can be obtained by comparing the actual quantitative value in fish with the regulatory value. It has also been reported that cooking reduces mercury levels in fish (45). Labeling of the heavy metal content of fish is considered necessary to enable consumers to select foods and cooking methods and control intake by considering risks and benefits (46).

To evaluate the performance of the LAEP-OES method, we compared it to the official method (CV-AAS). Various fish specimens were analyzed for comparison (Table 5). A high correlation was observed between the LAEP-OES method and the CV-AAS method (Spearman’s rank correlation coefficient, *r_s_* = 0.9849, *P *<0.0001, Figure 3). The median value obtained for the LAEP-OES method was higher than that for CV-AAS (0.67 versus 0.57, respectively, *P *<0.0001, Wilcoxon matched-pairs signed rank test, Supplemental Table 1). This difference in sensitivity to mercury values between the two methods was likely due to differences in protocols and facilities. Another reason for the higher sensitivity to mercury in the fish matrix may be that amino acids such as cysteine are contained in the solution. Cysteine is known to stabilize mercury (47), and it may contribute to the efficiency of concentrating it near the electrode. In fact, the LAEP-OES method for inorganic mercury has significantly low sensitivity in aqueous solution, but the same reason could be why the sensitivity was extremely high in the matrix. The influence of the matrix can be suppressed by creating a calibration curve using a solution containing cysteine. After correcting the slope to 1, no difference was observed in the mercury value distribution between the LAEP-OES method and the CV-AAS method. This suggests that the proposed method can generate comparable results to existing methods by correcting the slope. The LAEP-OES method for measuring mercury has potential application not only for bluefin tuna but also for other fish.

This system can be easily used without any special expertise. Simply by placing the single-use measurement reagent pack in the system, dilution, concentration, emission, and measurement can all be performed automatically. No complicated pretreatment of fish specimens is required. The total mercury concentration in fish can be determined 15 min after specimen processing. This system is also maintenance-free since it uses the single-use measurement reagent pack. This analytical method can be used for on-site monitoring of total mercury in fish.

## Conclusions

The LAEP-OES method for total mercury levels was validated using standard solutions, certified substances, and tuna specimens. The method had a strong positive correlation with CV-AAS. This method, which enables on-site measurements, will be useful for measuring and monitoring heavy metals in fish.

## CRediT Author Statement

Conceptualization, H.T., Y.S., and M.Y.; methodology, H.T. and M.Y.; software, H.T.; validation, H.T.; formal analysis, H.T.; investigation, H.T.; resources, H.T. and S.Y.; data curation, H.T. and S.Y.; writing—original draft preparation, H.T.; writing—review and editing, S.Y. and M.Y.; visualization, H.T.; supervision, S.Y.; project administration, S.Y. and M.Y.; funding acquisition, not applicable.

## Supplementary Material

qsae053_Supplementary_Data
