# Supplementary material for: Validation of a New Liquid Asymmetric-Electrode Plasma Optical Emission Spectroscopy (LAEP-OES) Method for Measurement of Total Mercury in Tuna
Source: J AOAC Int. 2024 Jun 28;107(6):943–52. doi: 10.1093/jaoacint/qsae053 (PMC11532634; doi:10.1093/jaoacint/qsae053)
Supplement: qsae053_Supplementary_Data [file qsae053_supplementary_data.zip › qsae053_Supplementary_Data/aoac-24-0130-File010.docx]

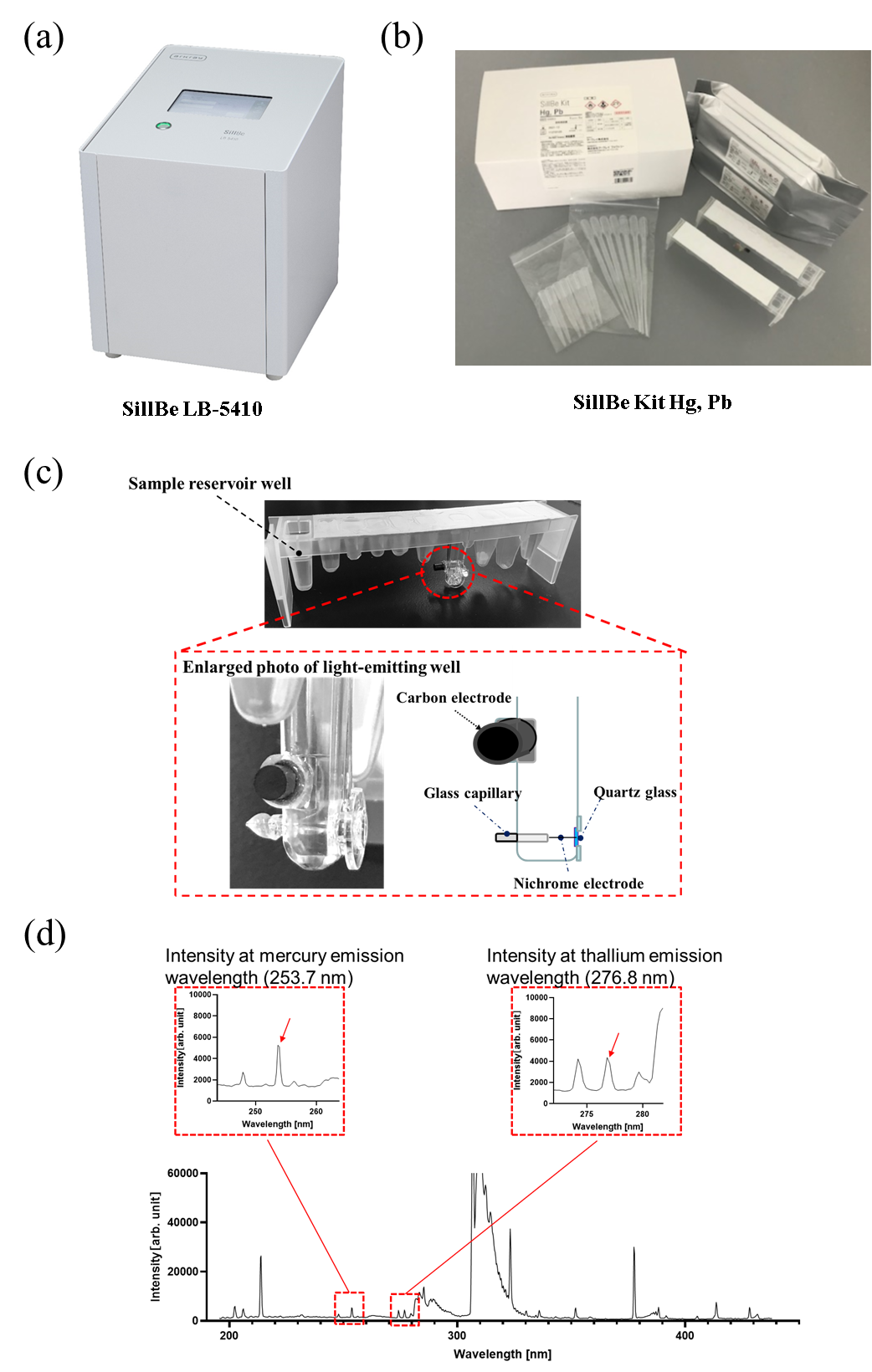


**Supplementary Figure 1.** **Description of the metal measurement system used in this study. (a)** Photograph of SillBe LB-5410. The single-use measurement reagent pack is placed in this device for measurement. **(b)** Photograph of SillBe Kit Hg, Pb. The kit includes supplies for six measurements: six single-use reagent packs for measuring heavy metals (mercury and lead) in human urine, seven single-use droppers, and seven single-use tips for use with a SillBe LB-5410 nozzle. **(c)** Photograph and structural diagram of a single-use measurement reagent pack for plasma optical emission spectrometry. The enlarged photograph shows a light-emitting well that has two types of electrodes: a carbon electrode (anode) and a nichrome electrode (cathode). **(d)** A representative spectrum of tuna lysate. Emission signals ranging from 190 to 450 nm are displayed. Square frames outlined with red dotted lines indicate the targets used in this research. Red arrows indicate spectral peaks used. Thallium is an internal standard reagent added to samples during measurements.
