# Supplementary material for: Validation of a New Liquid Asymmetric-Electrode Plasma Optical Emission Spectroscopy (LAEP-OES) Method for Measurement of Total Mercury in Tuna
Source: J AOAC Int. 2024 Jun 28;107(6):943–52. doi: 10.1093/jaoacint/qsae053 (PMC11532634; doi:10.1093/jaoacint/qsae053)
Supplement: qsae053_Supplementary_Data [file qsae053_supplementary_data.zip › qsae053_Supplementary_Data/aoac-24-0130-File011.docx]

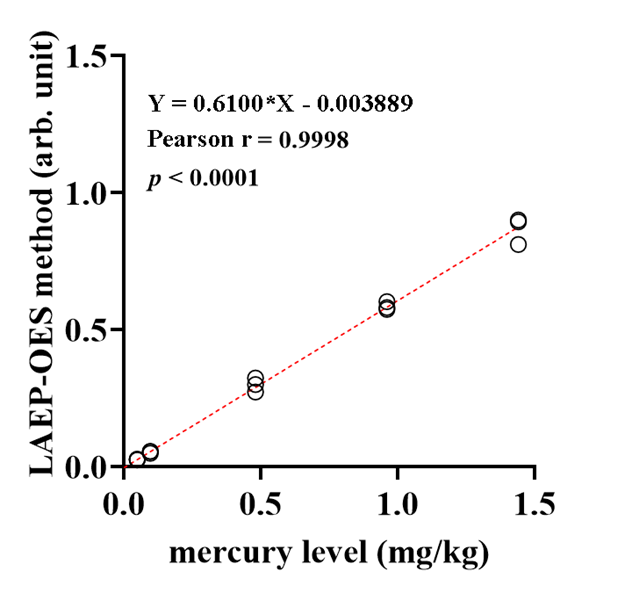


**Supplementary Figure 2.** **Calibration curve to determine mercury concentration.** The X-axis indicates mercury concentration in a methylmercury standard solution. Each methylmercury standard solution was produced by dilution of a 1000 mg/mL methylmercury solution with 4 M lithium hydroxide. The Y-axis indicates the value calculated from the obtained mercury signal intensity, thallium signal intensity and their baseline intensity. The details are as follows. The concentration was calculated by using the ratio of the signal intensity at the mercury-specific wavelength to the baseline signal intensity and subtracting by 1 (mercury signal intensity/baseline intensity - 1). Similarly, for thallium added as an internal standard, the ratio of the signal intensity at the thallium-specific wavelength to the baseline signal intensity is calculated and subtracted by 1 (thallium signal intensity/baseline intensity - 1). Finally, the ratio of the mercury ratio to the thallium ratio was calculated, which was plotted as the value for each mercury concentration. The regression line equation was used to calculate the mercury concentrations in this study (R^2^　= 0.9961, Slope = 0.6100, Y intercept = -0.003889, p < 0.0001).
